# A coronary artery disease-associated SNP rs6903956 contributed to asymptomatic hyperuricemia susceptibility in Han Chinese

**DOI:** 10.1186/s12944-015-0026-1

**Published:** 2015-04-19

**Authors:** Jianfen Meng, Wenfeng Tan, Yujing Zhu, Fang Wang, Xinli Li, Miaojia Zhang

**Affiliations:** Department of Rheumatology, the First Affiliated Hospital of Nanjing Medical University, Nanjing, Jiangsu China; Department of Cardiology, the First Affiliated Hospital of Nanjing Medical University, Nanjing, Jiangsu China; Current affiliation: Department of Rheumatology, the Fourth Affiliated Hospital of Nantong Medical College, Yancheng, Jiangsu China

**Keywords:** Asymptomatic hyperuricemia, Coronary artery disease, SNP rs6903956

## Abstract

**Background:**

To investigate the association of a coronary artery disease (CAD) risk SNP rs6903956 with asymptomatic hyperuricemia (aHU) susceptibility in Han Chinese.

**Methods:**

Two hundred and twenty one patients with aHU and 447 healthy controls were recruited for this study. SNP rs6903956 were genotyped using TaqMan probe.

**Results:**

The overall genotype and allele frequency distribution of the rs6903956 showed significant difference between aHU cases and controls (p <0.001 for genotype and allele, respectively). AA genotype of rs6903956 was significantly associated with aHU (OR = 8.672, 95% CI 2.811-26.753, *p* <0.001) in our Han Chinese aHU cohort. Multivariate logistic regression analysis indicated that rs6903956 might be an independent risk factor for aHU susceptibility (OR = 10.642 [2.671- 42.400], *p* = 0.001 for codominant model and OR = 9.205 [2.336-36.280], *p* = 0.002 for recessive model) after adjustment for some well- known CAD risk factors including age, gender, body mass index, smoking, hypertension, diabetes mellitus, abnormal glycometabolism, lipid abnormality and alcohol intake. No significant genotype-specific difference in uric acid levels was observed in aHU patients and controls.

**Conclusions:**

Our findings are the first to establish a genetic link of a CAD-associated rs6903956 with aHU in a Han Chinese population, providing the genetic evidence to support the close relationship between hyperuricemia and CAD.

## Background

Hyperuricemia (HU) is present in 5-30% of the general population and has been increasing around the world probably due to prolonged life expectancy, lifestyle and dietary changes, and the usage of certain drugs [1]. HU is the most significant risk factor for gout definitely, but still plays a controversial role in the development of several chronic diseases including coronary artery disease (CAD) [2,3].

Among them, the relation between HU and CAD has been a topic of much interest in worldwide recently. The growing numbers of epidemiologic and experimental evidence have suggested that elevated level of serum uric is an independent risk factor for CAD incidence and mortality [4]. Although how the HU triggers the development and progression of CAD remains unclear, endothelial cell dysfunction, vascular smooth muscle cell proliferation, platelet adhesiveness and renin-angiotensin system activation caused by high levels of uric acid in circulation might be the explanation [5].

In addition to dietary and deficiency of the enzyme uricase, the varying levels of uric acid in human population are influenced by genetic factors. To date, Genome-Wide association (GWA) studies have identified and replicated 28 genetic loci that are strongly associated with serum uric acid concentrations in European ancestry including 18 new regions in or near *TRIM46, INHBB, SFMBT1, TMEM171, VEGFA, BAZ1B, PRKAG2, STC1, HNF4G, A1CF, ATXN2, UBE2Q2, IGF1R, etc.*[6]. Associations for many of the 28 loci mentioned above such as *ABCG2, PDZK1, SLC16A9, SLC2A9,SLC22A11, SLC22A12* etc. were of similar magnitude in individuals of non-European ancestry [7-9]. In addition, recent case–control studies found that SNP rs1333049 on chromosome 9p21, *SLC2A9* and *hURAT1* contributed to the HU susceptibility in Han Chinese populations [10-12]. Interestingly, genetic variations in multiple HU risk genes included *ABCG2, PDZK1, SLC22A12* have also been linked to plasma lipoprotein (a) levels or hypertension, two most significant known risk factors for CAD [13-15]. These findings lead us to suspect that HU or dyslipidemia may at least partly share the common genetic background with CAD.

Recently, GWAS study identified that a novel strong risk locus of rs6903956 in *C6orf105* contributed to CAD susceptibility in Han Chinese (*p* = 4.87 × 10^−12^, odds ratio = 1.51) [16]. We therefore test whether this CAD-associated SNP is also a genetic signal for HU risk in our Chinese Han cohort.

## Results

### Characteristic of aHU cases and controls samples

The clinical features of individuals recruited in the study are summarized in Table 1. aHU patients had significantly increased proportions of males compared with controls. The frequency of abnormalities in serum levels of total cholesterol (TC), triglyceride (TG), blood glucose, BMI (Body Mass Index) and hypertension are higher in aHU than those in controls (Table 1, *p* < 0.05). Age and C-reactive protein was equally distributed between the two groups (Table 1).

**Table 1:** **Demographic and clinical characteristics of the study population**

**Category**		**Cases (n = 221)**		**Controls (n = 446)**		***p*** ^**a**^
		**n**	**%**	**n**	**%**	
Age (mean ± SD)		51.9 ± 13.6		51.3 ± 12.5		0.544
Gender	Males	158	71.5	184	41.3	<0.001
	Females	63	28.5	262	58.7	
BMI(mean ± SD)		26.2 ± 3.3		24.5 ± 3.2		<0.001
Smoking	Yes	105	47.5	124	27.8	<0.001
	No	116	52.5	322	72.2	
Drinking	Yes	76	34.4	67	15.0	<0.001
	No	145	65.6	379	85.0	
Hypertension	Yes	89	40.3	56	12.6	<0.001
	No	132	59.7	390	87.4	
Diabetes mellitus	Yes	3	1.4	0	0.0	0.036
	No	218	98.6	446	100.0	
Abnormal glycometabolism	Yes	77	34.8	29	6.5	<0.001
	No	144	65.2	417	93.5	
Lipid abnormality	Yes	99	44.8	3	0.7	< 0.001
	No	122	55.2	443	99.3	
C-reactive protein (mg/L)		4.04 ± 4.38		3.84 ± 3.26		0.527
Uric acid level (μmol/L)		467.4 ± 56.9		269.6 ± 56.2		< 0.001

^a^continuous variables were evaluated using Student’s t-test; categorical variables were Chi-square test.

### Association of rs6903956 with aHU susceptibility

In both patient and control groups, genotype and allele frequencies did not deviate significantly from those expected from the Hardy–Weinberg equilibrium. The allelic and genotypic frequencies of rs6903956 are shown in Table 2. Compared with healthy controls, aHU have a higher allelic frequency of A allele (0.176 versus 0.068, *p* <0.001). aHU also have a higher genotypic frequency for the homozygous AA genotype than healthy controls (0.063 versus 0.009, *p* <0.001). In codominant, dominant and recessive model, rs6903956 AA genotype was significantly associated with aHU susceptibility in our cohort (OR = 8.672 [2.811-26.753], *p* <0.001 for codominant model; OR = 2.782 [1.861-4.159], *p* <0.001 for dominant model; OR = 7.473 [2.430-22.983], *p* <0.001 for recessive model) (Table 3).

**Table 2:** **Genotypic and allelic frequencies of SNP rs6903956 in aHU cases and controls**

**Group**	**Genotype (%)**				**Allele (%)**			
	**GG**	**GA**	**AA**	***p***	**G**	**A**	**OR (95% CI)**	***p***
Cases	157(71.1)	50(22.6)	14(6.3)	<0.001	364(82.4)	78(17.6)	2.919(2.043-4.171)	<0.001
Controls	389(87.2)	53(11.9)	4(0.9)		831(93.2)	61(6.8)

**Table 3:** **The association of SNP rs6903956 genotype with aHU**

**Model**	**Genotype**	**Cases (n = 221)**		**Controls (n = 446)**		**OR (95% CI)** ^**a**^	***p*** ^**a**^	**Adjusted OR(95% CI)** ^**b**^	***p*** ^**b**^
		**n**	**%**	**n**	**%**				
codominant	GG	157	71.1	389	87.2	1.000 (Reference)		1.000 (Reference)	
	GA	50	22.6	53	11.9	**2.337(1.523-3.588)**	**<0.001**	**2.411(1.321-4.402)**	**0.004**
	AA	14	6.3	4	0.9	**8.672 (2.811-26.753)**	**<0.001**	**10.642(2.671-42.400)**	**0.001**
dominant	GG	157	71.0	389	87.2	1.000 (Reference)		1.000 (Reference)	
	GA + AA	64	29.0	57	12.8	**2.782(1.861-4.159)**	**<0.001**	**2.988(1.717-5.202)**	**<0.001**
recessive	GG + GA	207	93.7	442	99.1	1.000 (Reference)		1.000 (Reference)	
	AA	14	6.3	4	0.9	**7.473(2.430-22.983)**	**<0.001**	**9.205 (2.336-36.280)**	**0.002**

^a^using univariate logistic regression analysis; ^b^adjusted for age, gender, BMI, smoking, hypertension, diabetes mellitus, abnormal glycometabolism, lipid abnormality, drinking. Odds ratio (95% confidence interval) was expressed for the risk of the other genotype when GG or GG+GA genotype was referenced.

Since aHU patients had higher frequency of abnormality in serum levels of TGs, TC and blood glucose compared to controls in our patients, multivariate logistic regression analysis was used to exclude the effect of confounding factors on genetic association. rs6903956 AA genotype still conferred the strong association with aHU in our samples after adjustment for those well-known CAD risk factors including age, gender, BMI, smoking, hypertension, diabetes mellitus, abnormal glycometabolism, lipid abnormality and alcohol intake, suggesting rs6903956 might be an independent risk factor for aHU susceptibility (Table 3).

### Correlating SNPs rs6903956 and uric acid levels

**Figure 1:**
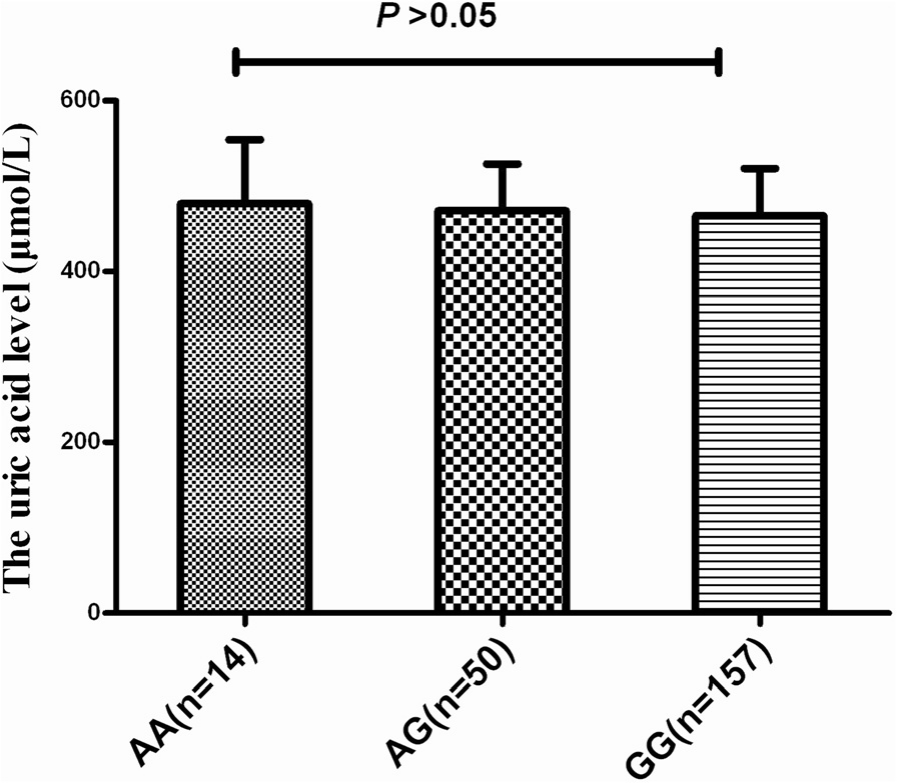
The uric acid levels of genotype of rs6903956 in cases. There is no difference on uric acid levels among the three genotypes of rs6903956 using ANOVA (479.4 ± 75.5 μmol/L for AA-carriers vs. 471.2 ± 55.0 μmol/L for AG-carriers, vs. 465.1 ± 55.9 μmol/L for GG-carriers, *p* > 0.05).

**Figure 2:**
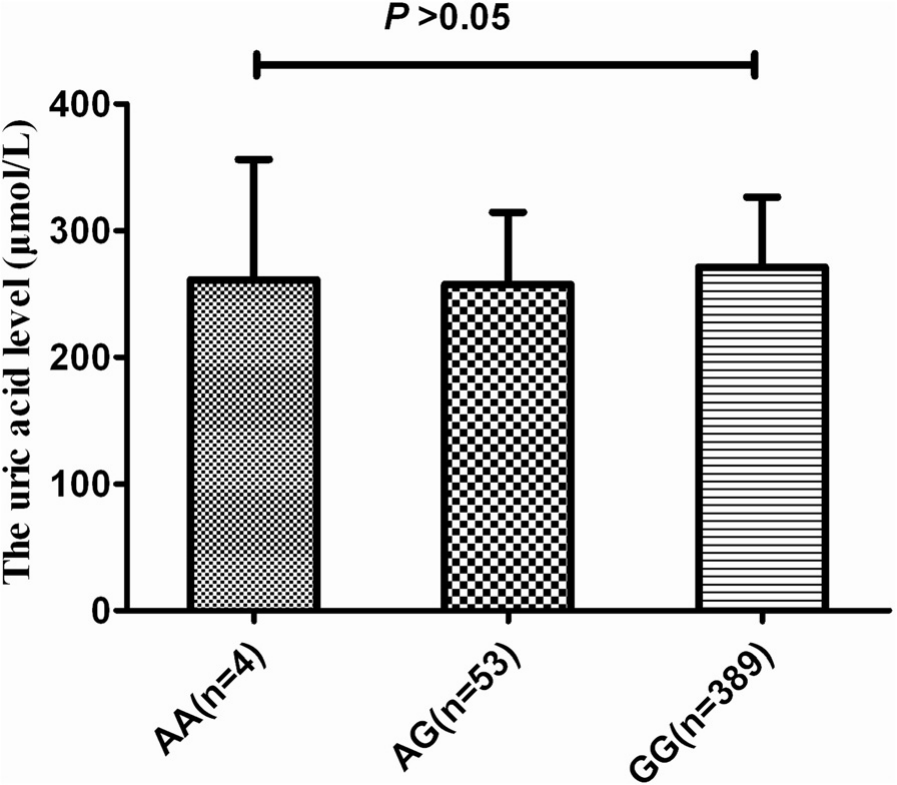
The uric acid levels of genotype of rs6903956 in controls. There is no difference on uric acid levels among the three genotypes of rs6903956 using ANOVA (261.3 ± 95.2 μmol/L for AA-carriers vs. 257.5 ± 57.2 μmol/L for AG-carriers, vs. 271.3 ± 55.5 μmol/L for GG-carriers, *p* > 0.05).

We further address whether genetic variation of rs6903956 could influence uric acid levels. No significant genotype-specific difference in uric acid levels was observed in aHU patients (Figure 1) and healthy controls (Figure 2), which might be attributed to the narrow range of uric acid levels in our study population (median = 453.6 μmol/L; 25% to 75% percentile = 431.5 μmol/L to 494.9 μmol/L in aHU, median = 264.9 μmol/L; 25% to 75% percentile = 224.5 μmol/L to 308.75 μmol/L in controls) or the insufficient power.

## Discussion

Elevated levels of serum uric acid have been suggested as an independent risk factor for CAD [17]. Previously GWAS study has confirmed the genetic association of rs6903956 with CAD in Han Chinese population [16]. We suspect that loci linked to CAD may also contribute to the pathogenesis of hyperuricemia or gout. We tested the association of this CAD-associated locus with aHU in present study. Our data are first to indicate that rs6903956 is contributed to aHU susceptibly in our Chinese Han population, providing the potential genetic evidence to support the close relationship between serum uric acid and CAD.

Considering these aHU patients have higher proportions of dyslipidemia, hyperglycemia and overweight than those in controls [18], it is important to clarify whether this locus is a real genetic signal for aHU or just is linked to certain of CAD risk factor. By multivariate logistic regression analysis, our data showed that AA genotype still remained the strong association with HU after adjustment for some well-known CAD risk factors included age, gender, BMI, smoking, blood pressure, blood glucose, cholesterol and alcohol consumption, suggesting rs6903956 might be an independent risk factor for aHU susceptibility. Similarly, Wang *et al.* reported a CAD-associated SNP rs1333049 at 9p21 is linked with gout susceptibility [10], which supported our data that hyperuricemia might at least partly share the common genetic background with CAD.

The rs6903956 is located in intron 1 of *C6orf105. C6orf105* is mainly expressed in heart, stomach, skin, kidney, endothelia cell and leukocytes and its function remains unclear. Interestingly, recently study found that *C6orf105* could encode a novel uncharacterized protein ADTRP to regulate androgen-enhanced tissue factor pathway inhibitor (TFPI) expression in cultured endothelial cells. TFPI is a key natural inhibitor of coagulation and play an important role in maintaining normal blood flow and endothelial cell function [19]. The deficiency of TFPI could promote atherosclerosis and thrombosis in mice highlighted a protecting role of TFPI in the pathogenesis of CAD [20]. Given the minor risk allele A of rs6903956 has been suggested to confer a decreased *C6orf105* mRNA expression in CAD patients [16], it is possible that genetic variant of *C6orf105* hence affect *TFPI* expression by altering ADTRP, resulting in endothelial cell dysfunction and abnormality of coagulation, which might explain rs6903956 contribute to CAD susceptibility. However, the mechanisms involved in the association of rs6903956 and aHU remains unclear. The heritability of serum urate concentrations is estimated at 40 − 70% [21]. Previous GWAS have so far identified more than 20 genomic loci associated with urate concentrations and gout, which explained about 5–6% of variance in serum urate concentrations [6]. Unfortunately, we failed to find a genetic association of rs6903956 with uric acid levels, the narrow range of uric acid levels in our study population might be an explanation. Further studies are needed to clarify the real role of C6orf105 on uric acid production and transport.

Half of aHU patients in current study are >52 years who had been exposed to a greater cardiovascular risk but still lack of any clinical symptom of CAD, supporting that these patients are a good asymptomatic hyperuricemia cohort to study the genetic link between HU and CAD; however, we could not exclude the possibility that some subclinical CAD patients were still mixed in this study. Therefore, further prospective studies in a larger population with longer follow-up are needed to confirm the genetic link between HU and CAD and the potential role of this SNP in acid homeostasis.

## Methods

### Patients and controls

To explore the potential genetic link between HU and CAD, a total of 221 asymptomatic hyperuricemia (aHU) patients and 447 health volunteers from Gaoyou rural district of Jiangsu Province, China were recruited for this study. aHU were defined as: 1) people with serum uric acid levels >420 μmol/L in man and post-menopausal woman, and as >350 μmol/L in premenopausal woman; 2) these patients never suffered from gout, and no self-reported history of CAD and/or any symptom and sign of CAD after carefully physical examination and medical history inquiry [10]. Healthy controls were defined as individuals without hyperuricemia, CAD and significant abnormalities in plasma glucose, total cholesterol (TC) and triglycerides (TG) levels. The smoker was defined as people who consumed at least one cigarette a day for more than a year. Drinking was defined as those who reported frequent or daily alcohol consumption. Abnormal glycometabolism was defined as fasting blood glucose ≥6.0 mmol/L. Lipid abnormality was defined as abnormal levels of more than one of the following lipid fraction: triglycerides ≥2.25 mmol/L, total cholesterol ≥6.25 mmol/L, high density lipoprotein ≤0.9 mmol/L, low density lipoprotein ≥4.11 mmol/ L. Persons with hypertension history and/or receiving anti-hypertensive medication were considered hypertensive. Individuals with diabetes mellitus history and/or use hypoglycemic medications were defined as diabetes mellitus. CAD was defined by the self-reported history and/or receiving the treatment of CAD. The Ethics committee of the First Affiliated Hospital of Nanjing Medical University approved the study protocol and informed consent was obtained from each of the eligible participants before recruitment. The study was in accordance with the principles of the current version of the Declaration of Helsinki. All participants were measured and recorded for body mass index (BMI), plasma levels of blood glucose, creatinine, uric acid, TC and TG. All biochemical analysis was performed in the central laboratory of the First Affiliated Hospital of Nanjing Medical University.

### Genotyping

Genomic DNA was obtained from the peripheral blood leukocytes using the PureGene DNA Blood Kit (QIAGEN, Germany). The SNP rs6903956 was genotyped by TaqMan probe (ABI Assay ID: 4351379; Applied Biosystems, Foster City, CA, USA). The reaction mix was made from 20 × SNP genotyping assay, 2 × TaqMan Genotyping Master Mix, no AmpErase UNG, and DNA-free water. The final reaction volume per well is 5 μL containing 1 × SNP genotyping assay, 1 × TaqMan Genotyping Master Mix and about 20 ng DNA for a 384-well plate. The PCR reaction was performed according to the manufacturer’s protocol using the Applied Biosystems 7900HT Fast Real-Time PCR System: Enzyme Activation at 95°C for 10 minutes; denature at 95°C for 15 seconds, extension at 60°C for 1 minute, a total of 40 cycles. Post-PCR plate read and analysis were performed by Sequence Detection System software version 2.4 (Applied Biosystems).

### Statistical analysis

Genotypes were tested for Hardy-Weinberg equilibrium among all participants using a Chi-square test. Allele and genotype frequencies of rs6903956 were obtained by direct counting and the distributions comparison was performed by the Chi-square test. Differences in selected demographic continuous variables among groups were evaluated by one-way analysis of variance (ANOVA) or the Kruskal-Wallis H test. Categorical variables including sex, smoking status, alcohol consumption status, hypertension status, diabetes status were compared among the genotypes of rs6903956 using a Chi-square test. The association of rs6903956 genotype and aHU risk were estimated using codominant model (compared three genotype), dominant model (defined as GG vs. GA + AA) and recessive model (defined as GG + GA vs. AA), respectively. Odds ratios (OR) with their 95% confidence intervals (CI) were reported. A multivariate logistic regression analysis was used for association analyses with adjustments for age, gender, BMI, smoking, hypertension, and diabetes mellitus. P-value < 0.05 was considered statistically significant.

The statistical power calculation for the case–control analysis was performed using Quanto software (http://hydra.usc.edu/gxe/_vti_bin/shtml.dll/request.htm). Considering rs6903956 minor allele frequency of 10% that was obtained in Han Chinese CAD patients [16], our sample size had 90% power (α = 0.05) to detect genetic effects with an OR ≥1.5 (estimated hyperuricemia prevalence in the background population = 5-30%).
